# Sex-Specific Differences in Metabolic Syndrome Classification Using IDF Versus NCEP-ATP III Criteria: A Systematic Review and Head-to-Head Meta-Analysis

**DOI:** 10.3390/metabo16100701

**Published:** 2026-09-22

**Authors:** Phuong Minh Bui Thi, Tien Van Nguyen, Dat Minh Le

**Affiliations:** 1 Department of Biochemistry, Thai Binh University of Medicine and Pharmacy, Tran Lam Ward, Hung Yen 06122, Vietnam; minhphuongytb@gmail.com; 2 Department of Health Organization and Management, Thai Binh University of Medicine and Pharmacy, Tran Lam Ward, Hung Yen 06122, Vietnam; 3 Vietnam Public Health Association, Dong Da Ward, Hanoi 11527, Vietnam; minhdat.k6.yhdp@gmail.com

**Keywords:** metabolic syndrome, International Diabetes Federation, NCEP-ATP III, sex differences, prevalence, classification, systematic review, meta-analysis

## Abstract

**Background/Objectives**: Metabolic syndrome (MetS) classification varies between the International Diabetes Federation (IDF) and National Cholesterol Education Program Adult Treatment Panel III (NCEP-ATP III) definitions. We compared their same-sample classification yield by sex. **Methods**: PubMed/MEDLINE and Scopus were searched from database inception through 30 August 2026, with reference-list screening and citation tracking. Eligible observational studies assessed both definitions in the same adult sample; intervention-effect reports without an eligible observational comparison were outside the scope. One reviewer performed selection, extraction, and JBI-based appraisal. Random-effects models pooled sex-specific prevalence and prevalence ratios; revised ATP III was examined in sensitivity analysis. The review was not registered. **Results**: Seven studies (95,887 participants) were included; five (74,399 participants; 36,033 men and 38,366 women) contributed to the primary meta-analysis. The pooled IDF/NCEP-ATP III prevalence ratio was 0.95 (95% CI 0.65–1.37) in men and 1.15 (1.01–1.31) in women. Sensitivity estimates were 0.91 (0.70–1.19) and 1.08 (0.92–1.27), respectively. Heterogeneity was extreme (I^2^ 92.3–99.9%). Three primary studies were conducted in Asian countries and represented 89.1% of primary-analysis participants. **Conclusions**: No clear pooled difference was observed in men; the modest female excess under IDF attenuated in sensitivity analysis. Five independent studies, extreme heterogeneity, and geographically concentrated samples limit robustness and generalizability. The findings provide a hypothesis-generating evidence summary, not proof of diagnostic equivalence, biological mechanisms, prognostic superiority, or treatment benefit. Definition-specific and sex-stratified reporting is warranted. No external funding was received.

## 1. Introduction

Metabolic syndrome (MetS) describes the clustering of abdominal obesity, hyperglycemia, elevated blood pressure, hypertriglyceridemia, and reduced high-density lipoprotein cholesterol (HDL-C), and is widely used to identify an adverse cardiometabolic profile. However, MetS is not defined uniformly. The National Cholesterol Education Program Adult Treatment Panel III (NCEP-ATP III) and International Diabetes Federation (IDF) definitions differ in their diagnostic rule and some component thresholds [1,2].

The definitions compared here arose from distinct conceptual and clinical priorities. Reaven’s 1988 Banting lecture linked insulin resistance to a cluster of metabolic and cardiovascular abnormalities, providing a pathophysiological framework rather than the later five-component diagnostic algorithm [3]. The provisional WHO report in 1998 and the WHO consultation report in 1999 operationalized MetS around impaired glucose regulation, diabetes, and/or insulin resistance plus additional abnormalities, including microalbuminuria [4,5]. NCEP-ATP III subsequently emphasized a practical combination of routinely measured risk factors rather than mandatory demonstration of insulin resistance [1].

The original 2001 NCEP-ATP III definition requires any three of five abnormalities: waist circumference > 102 cm in men or >88 cm in women; triglycerides ≥ 150 mg/dL (1.7 mmol/L); HDL-C < 40 mg/dL (1.03 mmol/L) in men or <50 mg/dL (1.29 mmol/L) in women; systolic blood pressure ≥ 130 mmHg or diastolic blood pressure ≥ 85 mmHg; and fasting glucose ≥ 110 mg/dL (6.1 mmol/L). Central obesity is one component rather than a mandatory condition [1].

The IDF consensus definition requires central obesity plus any two of four abnormalities: triglycerides ≥ 150 mg/dL or specific treatment; HDL-C < 40 mg/dL in men or <50 mg/dL in women, or specific treatment; systolic blood pressure ≥130 mmHg or diastolic blood pressure ≥ 85 mmHg, or treatment for previously diagnosed hypertension; and fasting plasma glucose ≥ 100 mg/dL (5.6 mmol/L) or previously diagnosed type 2 diabetes. Waist thresholds are sex- and ethnic-group-specific: for example, ≥94/80 cm in Europid men/women and ≥90/80 cm in South Asian and Chinese men/women. Central obesity may be assumed when body mass index is >30 kg/m^2^ [2,6].

In parallel with the IDF definition, the 2005 American Heart Association/National Heart, Lung, and Blood Institute (AHA/NHLBI) statement retained the ATP III any-three-of-five framework, adopted a fasting glucose threshold of 100 mg/dL, and recognized treatment for specified abnormalities [7]. The 2009 joint interim statement sought harmonization by removing compulsory central obesity while retaining population- or country-specific waist thresholds [8]. These milestones reflect changing operational choices, not a demonstrated sequence of progressively more accurate diagnostic tests.

Supplementary File S1 provides the characteristics and roles of the included studies (Table S1), domain-level risk-of-bias assessments (Table S2), study-level sex-specific prevalence estimates (Table S3), study-level head-to-head prevalence ratios (Table S4), and a summary of the primary and sensitivity syntheses (Table S5). Supplementary File S2 provides the available search syntax and documentation status (Table S6), eligibility criteria and report dispositions (Table S7), geographic representation (Table S8), and selected historical milestones in the development of metabolic syndrome definitions (Table S9). These are reference definitions, not an assumption that every publication bearing an ATP III label used identical thresholds. Changes in waist and glucose thresholds, medication rules, and the requirement for central obesity can alter the direction and the magnitude of a head-to-head comparison. Selected historical milestones and their relevance to this review are summarized in Supplementary File S2, Table S9. This review retained the distinction between the primary NCEP-ATP III comparison and the explicitly revised ATP III sensitivity comparison; no individual participants were reclassified from raw measurements.

Sex-specific differences in body-fat distribution, waist circumference, menopausal transition, glucose metabolism, and cardiovascular risk profiles complicate interpretation of MetS prevalence. In the Neyshabur Longitudinal Study on Ageing, older women had markedly higher MetS prevalence than men under both ATP III- and IDF-based definitions. In contrast, a large Vietnamese workforce study found higher prevalence in men at younger ages but a steeper age-related increase among women [9,10].

Primary studies comparing the two definitions have also reported inconsistent outcome-related findings. An Iranian analysis found that NCEP-ATP III aligned more strongly than IDF with categories of estimated atherosclerotic cardiovascular disease (ASCVD) risk, despite IDF identifying more cases [11]. In a prospective Chinese cohort, revised ATP III showed the highest sensitivity and shortest receiver operating characteristic (ROC) curve distance for incident cardiovascular disease, whereas IDF showed higher specificity in men [12]. Population studies from Finland, Pakistan, and Sierra Leone likewise reported definition-dependent prevalence and variable agreement [13,14,15].

Most available comparisons are population-specific, and comparisons across unrelated samples cannot separate criterion-related differences from differences in age structure, ethnicity, setting, and cardiometabolic burden. A head-to-head synthesis restricted to studies applying both definitions within the same sample can reduce this source of confounding. Therefore, this systematic review and meta-analysis compared sex-specific MetS classification under IDF and NCEP-ATP III, with secondary evaluation of criterion-specific prevalence, agreement, and downstream outcome-related evidence. Its contribution is a structured synthesis of classification differences and their uncertainty, not a new diagnostic framework, mechanistic validation, or evidence of improved treatment outcomes.

## 2. Materials and Methods

### 2.1. Design, Reporting, and Registration

This systematic review and meta-analysis was structured around same-sample comparisons of IDF and NCEP-ATP III and was reported with reference to the PRISMA 2020 statement [16]. The protocol was not prospectively registered. To reduce post hoc decision-making, the eligibility criteria, outcomes, and quantitative analysis plan were finalized before completion of the full-text review.

The historical overview in the Introduction and Supplementary Table S9 is a targeted narrative account of foundational and consensus publications added for context during revision. These contextual references were not added as eligible comparative studies and did not change the review population, PRISMA counts, or quantitative syntheses.

### 2.2. Eligibility Criteria

Original human observational studies were eligible when they (1) enrolled adults or older adults; (2) assessed IDF and NCEP-ATP III within the same underlying sample; (3) provided extractable overall or sex-specific prevalence, agreement measures, or associations with downstream cardiometabolic outcomes; and (4) were published in English or Vietnamese. Cross-sectional, cohort, and case–control designs were eligible. A previous clinical diagnosis of MetS was not required: eligibility concerned application of both classification systems to the source population, not enrollment exclusively of patients already known to have MetS.

The review addressed observational classification and association, not intervention effectiveness. Reports of dietary, exercise, pharmacological, surgical, or other interventions were not eligible merely because participants had MetS. An intervention-effect report without an eligible same-sample observational comparison of IDF and NCEP-ATP III was outside scope. The seven included reports contributed observational assessments, and no treatment effects or intervention-induced changes in MetS were pooled.

The exclusion criteria were: exclusively pediatric populations; absence of a direct or reconstructable IDF versus NCEP-ATP III comparison in the same sample; insufficient numerical information for any eligible review outcome; duplicate reporting of a dataset without additional eligible information; publication in a language other than English or Vietnamese; and reports that did not provide original eligible observational data, such as reviews, editorials, or intervention-effect reports alone. An Abstract-only report was excluded when its available information was insufficient to establish eligibility or extract the required data. A report whose full text could not be retrieved was recorded separately as not retrieved, rather than treated as a confirmed full-text eligibility exclusion.

Eligibility for the review was distinguished from eligibility for a particular synthesis. The strict primary meta-analysis additionally required the NCEP-ATP III comparator designated for that analysis and extractable sex-specific prevalence data. Reports with revised or modified ATP III-family comparators, or without the sex-specific information required for primary pooling, could contribute to sensitivity or narrative synthesis when they met the broader review criteria. Thus, exclusion from the primary meta-analysis did not necessarily mean exclusion from the systematic review. Supplementary File S2 (Table S7) summarizes these distinctions and the report-disposition information available for this revision.

### 2.3. Information Sources and Search Strategy

PubMed/MEDLINE (U.S. National Library of Medicine, Bethesda, MD, USA) and Scopus (Elsevier, Amsterdam, The Netherlands) were searched from database inception through 30 August 2026. This is the final search date used consistently in the Abstract, Methods, Results, and Figure 1’s legend. The search was not restricted to publications from 2021 onward; 2021–2024 describes the publication years of the included studies, not the intended search interval. Reference-list screening and citation tracking were reported as supplementary search methods. No unpublished data were sought.

The search combined four concept blocks: metabolic syndrome; International Diabetes Federation or IDF; NCEP-ATP III or ATP III; and comparison- or sex-related terms. The database-specific Boolean strings available for this revision are reproduced in Supplementary File S2 (Table S6), with the reported final date and documentation limitations. English/Vietnamese language and adult human populations were eligibility restrictions; the distinction between these restrictions and filters explicitly encoded in each supplied string is stated in Table S6. Database-specific yields and the original time-stamped search histories were not available for independent verification; the reported combined yield is therefore presented without inventing separate PubMed or Scopus counts.

### 2.4. Study Selection Process

Records were imported into a PRISMA screening log and deduplicated. One reviewer screened Titles and Abstracts against the eligibility criteria and assessed potentially relevant reports. Eligibility decisions were rechecked against the source articles before synthesis. Duplicate records removed before screening were distinguished from duplicate publications of the same dataset. Retrieval failures were recorded separately from eligibility exclusions. The available record of the two eligibility exclusions and one retrieval failure is summarized in Supplementary File S2 (Table S7); their bibliographic identities could not be reconstructed from the supplied materials, which limits independent audit of the selection process.

### 2.5. Data Extraction

A standardized extraction form was used to record first author, publication year, country, study design and setting, sample size, sex distribution, age definition, diagnostic framework, criterion-specific prevalence, sex-stratified estimates, agreement statistics, and downstream metabolic or cardiovascular outcomes. Where reported, sensitivity, specificity, predictive values, ROC-related indices, hazard ratios, odds ratios, and kappa coefficients were also extracted. One reviewer performed the extraction. When exact sex-specific case counts were not reported but sex-specific prevalences and denominators were available, counts were reconstructed by nearest-integer rounding for quantitative synthesis.

### 2.6. Outcomes

The primary outcome was the within-study prevalence ratio for MetS classification under IDF versus NCEP-ATP III, calculated separately for men and women. Secondary outcomes were pooled sex-specific prevalence under each definition, agreement between definitions, and criterion-specific associations with downstream cardiometabolic outcomes. Original NCEP-ATP III comparisons defined the strict primary synthesis; revised ATP III comparisons were reserved for sensitivity analysis.

### 2.7. Risk-of-Bias Assessment

Risk of bias was assessed using JBI-based appraisal domains appropriate to observational prevalence and association studies [17]. The assessment considered the sampling frame, participant recruitment, measurement of the IDF and NCEP-ATP III definitions, outcome ascertainment, control of confounding, and appropriateness of the statistical analysis. Domain-level judgments were derived from the full reports and summarized as low, moderate, or high overall risk.

### 2.8. Statistical Analysis

Quantitative synthesis was performed in R version 4.5.3 for Windows (R Foundation for Statistical Computing, Vienna, Austria) using random-effects models. For the strict primary synthesis, only studies using original IDF and NCEP-ATP III definitions and reporting extractable sex-specific prevalence data were included. Prevalence under each definition was pooled separately for men and women after logit transformation. For the head-to-head comparison, within-study prevalence ratios (IDF/NCEP-ATP III) were calculated by sex and synthesized on the logarithmic scale. Between-study heterogeneity was evaluated using Cochran’s Q, I^2^, and τ^2^. Because most reports did not provide paired cross-classification tables for the same participants, prevalence ratios were derived from marginal sex-specific prevalence estimates, and within-participant covariance could not be incorporated. A prespecified sensitivity analysis added one study using revised ATP III as an ATP III-family comparator. Given the small number of studies, subgroup analysis and meta-regression were not undertaken, and pooled prevalence estimates were treated as descriptive summaries rather than transportable population estimates.

### 2.9. Assessment of Small-Study Effects

Assessment of funnel-plot asymmetry and Egger’s regression was prespecified only for syntheses containing at least 10 studies. Because each quantitative synthesis included fewer than 10 studies, formal tests for publication bias or small-study effects were not performed.

## 3. Results

### 3.1. Study Selection

For the searches reported through 30 August 2026, 17 records were identified across PubMed/MEDLINE and Scopus. After removal of five duplicates, 12 records were screened by Title and Abstract, and two were excluded. Ten reports were sought for retrieval; one could not be retrieved. Nine reports were assessed for eligibility, and two were excluded: one lacked a direct same-sample IDF versus NCEP-ATP III comparison, and one was available only as a conference abstract without sufficient extractable information. Seven studies entered the systematic review. Five contributed to the strict primary meta-analysis, and one additional study contributed to the ATP III-family sensitivity analysis (Figure 1). These counts are retained from the supplied selection record and have not been independently regenerated from database exports for this revision.

### 3.2. Characteristics of Included Studies

The seven included studies were published between 2021 and 2024 and represented 95,887 participants from Vietnam, Iran, Pakistan, China, Finland, and Sierra Leone. Settings included occupational health screening, community-based population surveys, an ageing cohort, and an analytical cross-sectional risk study. Sample sizes ranged from 600 participants in the Iranian ASCVD-focused study to 57,997 participants in the Vietnamese workforce study [9,10,11,12,13,14,15]. Study characteristics are summarized in Table 1.

Three of the five primary studies were conducted in Asian countries (Vietnam, Iran, and Pakistan), comprising 66,318 of 74,399 participants (89.1%). The Vietnamese workforce study alone contributed 57,997 participants (78.0% of the primary-analysis sample). The remaining primary studies were from Finland (*n* = 5687) and Sierra Leone (*n* = 2394). These percentages describe participant representation, not random-effects meta-analysis weights or individual ethnicity. The Chinese study contributed to sensitivity and narrative analyses, and the second Iranian study contributed only to narrative synthesis. The study roles and denominators are provided in Supplementary File S1, Table S1, and summarized in Supplementary File S2, Table S8.

### 3.3. Risk of Bias

Most studies directly measured anthropometric, blood-pressure, and biochemical variables, which reduced misclassification of MetS components. Nevertheless, differences in sampling frame, participant recruitment, age structure, waist-circumference thresholds, and study setting affected representativeness and comparability. Overall risk of bias ranged from low to high: three studies were rated low risk, three moderate risk, and one high risk (Table 2).

Judgments were harmonized across observational designs and should be interpreted in the context of differences in study purpose and reporting detail.

### 3.4. Sex-Stratified Prevalence

Five studies using original IDF and NCEP-ATP III criteria and reporting extractable sex-specific prevalence data contributed to the strict primary synthesis, representing 74,399 participants (36,033 men and 38,366 women) [9,10,13,14,15]. Under IDF, the pooled prevalence of MetS was 21.6% (95% CI 8.7–44.3; I^2^ = 99.9%) in men and 24.8% (95% CI 7.8–56.2; I^2^ = 99.9%) in women. Under NCEP-ATP III, the corresponding pooled prevalence was 22.1% (95% CI 12.7–35.6; I^2^ = 99.7%) in men and 21.8% (95% CI 6.6–52.3; I^2^ = 99.9%) in women (Table 3 and Figure 2). The wide confidence intervals and extreme heterogeneity indicate that these pooled values should be interpreted as descriptive summaries of the included studies.

### 3.5. Head-to-Head Comparison and Sensitivity Analysis

Across the same five studies, the pooled IDF/NCEP-ATP III prevalence ratio was 0.95 (95% CI 0.65–1.37; I^2^ = 99.1%) in men and 1.15 (95% CI 1.01–1.31; I^2^ = 92.3%) in women (Figure 3). Thus, there was no clear overall difference in classification among men, whereas IDF classified modestly more women in the strict primary analysis. Individual study effects varied in direction, and the pooled estimates remained highly heterogeneous. When the study using revised ATP III was added, the corresponding ratios changed to 0.91 (95% CI 0.70–1.19) in men and 1.08 (95% CI 0.92–1.27) in women, attenuating the female difference and supporting cautious interpretation.

### 3.6. Agreement and Sex-Specific Patterns

Agreement between IDF and NCEP-ATP III varied from weak in the Iranian ASCVD-focused study to moderate or substantial in studies from Finland, Pakistan, Sierra Leone, and older Iranian adults [9,11,13,14,15]. Reported kappa values ranged from <0.20 to 0.79. Sex-specific patterns were likewise inconsistent. Women had higher reported prevalence in the Pakistani and older Iranian studies, whereas men had higher prevalence in the Finnish study under both definitions (Supplementary File S1, Table S3). In Vietnam, overall IDF prevalence was similar in men and women (8.5% and 8.3%), while NCEP-ATP III prevalence was higher in men (14.6% versus 6.1%). The Vietnamese report also described a steeper age-related increase among women, with female prevalence exceeding male prevalence after 60 years [9,10,14].

### 3.7. Downstream Outcome-Related Evidence

Outcome-related evidence was limited and not sufficiently homogeneous for meta-analysis. In Iran, NCEP-ATP III aligned more strongly than IDF with categories of estimated ASCVD risk [11]. In China, revised ATP III had the highest sensitivity and shortest ROC-curve distance for incident cardiovascular disease in both sexes, whereas IDF had higher specificity in men [12]. The Pakistani study further showed that associated factors and agreement varied across definitions within the same ostensibly healthy population [14]. These findings describe criterion-related differences but do not establish that either definition has universally superior prognostic performance.

## 4. Discussion

This review found that IDF and NCEP-ATP III did not classify MetS uniformly across populations or between sexes. The primary meta-analysis did not show a clear pooled difference in men; the confidence interval was also compatible with meaningful differences in either direction and does not establish equivalence. In the primary analysis, IDF classified a modestly higher proportion of women as having MetS than NCEP-ATP III, although the pooled prevalence ratio was close to 1. Individual study estimates varied in direction, and between-study heterogeneity was substantial (I^2^ > 90%). This difference in classification among women was attenuated when the additional study using revised ATP III was included in the sensitivity analysis. The contribution is a sex-stratified summary of the direction, magnitude, and uncertainty of same-sample classification differences, rather than a new demonstration of metabolic complexity. These findings provide a basis for further research, not validation of a universally superior definition.

The history of MetS definitions helps explain why classification disagreement is not, by itself, evidence that one system is erroneous. Emphasis shifted from insulin-resistance-centered concepts to practical combinations of risk factors and, later, harmonized rules that still allow variation in waist thresholds [1,2,3,4,5,6,7,8]. IDF requires central obesity, whereas NCEP-ATP III permits classification from three non-waist components; these structural differences can alter both the number and the phenotype of people classified. The present synthesis evaluates the consequences of such operational choices. It does not test whether MetS has a single biochemical cause or whether a later definition is necessarily more valid than an earlier one.

Extreme heterogeneity was a defining feature of the quantitative results. The included studies differed in age distribution, country, ethnicity, recruitment setting, occupational versus community sampling, waist-circumference thresholds, and baseline cardiometabolic burden. Accordingly, the pooled prevalence estimates should not be interpreted as global or directly transportable prevalence values. The within-study prevalence ratio more directly addressed the review question because each study applied both definitions to the same sample; nevertheless, this measure also varied markedly across settings and could not account for within-participant covariance when paired cross-classification tables were unavailable.

Only five independent studies contributed to each strict primary synthesis. The total of 74,399 participants improves within-study information but does not compensate for having only five study settings from which to estimate between-study variation. With so few studies, the pooled estimate and estimated heterogeneity may be sensitive to individual studies, model assumptions, and comparator classification. Random-effects pooling accommodates heterogeneity; it does not eliminate population differences or guarantee a stable estimate for a new setting. The very high I^2^ values and wide prevalence intervals reinforce the need to treat these estimates as descriptive rather than globally representative [18].

The female prevalence ratio illustrates this uncertainty: the primary estimate was 1.15 (95% CI 1.01–1.31), whereas the ATP III-family sensitivity estimate was 1.08 (0.92–1.27). Adding the revised comparator changes the evidence set and the comparator definition, so this is evidence of sensitivity to analytic scope, not proof of robustness under all plausible models. Similarly, a statistically detectable result in women and a non-detectable result in men do not themselves establish a sex-by-definition interaction; no formal interaction test was performed. We therefore do not infer a definitive female-specific advantage of IDF.

A five-study evidence base also limits investigation of heterogeneity and of selective publication. No regional subgroup analysis, meta-regression, or formal funnel-plot asymmetry test was undertaken. The absence of such tests must not be interpreted as absence of reporting bias. Restriction of primary pooling to reports with extractable sex-specific results may additionally favor better-reported studies. No new leave-one-out analysis or prediction interval has been presented in this revision; sensitivity to individual study omission and the expected range in a new population consequently remain unquantified. Additional independent studies and paired classification data are needed to assess robustness more fully.

Geographic concentration further limits generalizability. Vietnam, Iran, and Pakistan accounted for three of five primary studies and 89.1% of participants; the large Vietnamese study enrolled employees rather than a general population sample. This predominance concerns the composition of the evidence, not necessarily the statistical weight of those studies under random-effects pooling. Moreover, country of recruitment is not an individual-level measure of ethnicity, and populations within Asia are not homogeneous. Differences in age, body-fat distribution, metabolic risk profiles, recruitment setting, and the waist thresholds actually used could modify classification yield, but these mechanisms were not tested by this review.

IDF waist thresholds are ethnic-group-specific rather than simply country-specific [6]. A lower waist threshold can classify more individuals as centrally obese, whereas the requirement for central obesity can omit individuals who meet three non-waist ATP III components; therefore, the net direction of the IDF/NCEP-ATP III ratio cannot be predicted from an Asian setting alone. The supplied study-level results illustrate this variability: the Finnish ratios were 1.29 in men and 1.20 in women, whereas the Sierra Leonean ratios were 0.73 and 0.70, respectively. The Asian studies also differed in direction among men (0.58 in Vietnam, 0.90 in Iran, and 1.56 in Pakistan). These are descriptive within-study contrasts, not a statistical test of geographic or ethnic effect modification.

The size, direction, and sex pattern of the comparison could consequently differ in other populations, but the present data cannot quantify those differences. One Finnish study and one urban Sierra Leonean study are insufficient to represent Europe and sub-Saharan Africa, and no primary study represented the Americas or Oceania. The pooled estimates should not be used to select a preferred definition for those regions. Broader population-based comparisons, explicit reporting of ethnic composition and waist/glucose/medication rules, and preplanned interaction analyses are needed before extending these findings to underrepresented settings.

Population-related heterogeneity should not be equated with proof that a common framework is impossible. The 2009 joint interim statement illustrates that a shared diagnostic structure can coexist with population-specific waist thresholds [8]. Conversely, harmonization cannot be assumed to confer identical classification frequencies or predictive performance in every population. This review did not synthesize epigenetic measurements or test mediation by ancestry, environment, or lifestyle. Epigenetic explanations for the observed differences therefore remain hypotheses outside the available data; between-country comparisons cannot establish an epigenetic mechanism.

Operational standardization, prognostic validity, and clinical utility are distinct. A common classification rule can improve comparability of reporting, but superiority for prognosis requires prospective validation, and treatment benefit requires evidence that a management strategy improves patient outcomes. Only a small subset of the included studies evaluated cardiovascular outcomes or risk scores, with different comparators and endpoints [11,12]. No treatment effects were synthesized. Consequently, a higher classification yield does not establish better prediction or better care, and the present results do not support choosing IDF or NCEP-ATP III to direct or intensify treatment. The immediate implication is transparent reporting of the definition used, rather than a change in clinical management based on these pooled ratios.

For public health surveillance and comparative research, investigators should specify the exact MetS definition, component thresholds, medication rules, and any population adaptations. Reporting definitions in parallel, with sex- and age-stratified estimates and paired cross-classification tables, would permit more informative comparisons. Future cohorts should examine absolute event risks, discrimination, and calibration in concordantly and discordantly classified groups, using consistent measurements and prespecified analyses across populations. A separate question is whether classification-guided management improves outcomes; this requires comparative clinical-utility evidence, not prevalence comparisons alone. Studies addressing proposed biological explanations would need direct measurements, including epigenetic data where relevant, rather than attributing differences to country or ethnicity from aggregate estimates. These are priorities for further research, not findings demonstrated by this review.

This review prioritized same-sample comparisons, distinguished the primary comparator from the explicitly revised ATP III sensitivity comparison, and interpreted prevalence, agreement, and outcome-related evidence together. Important limitations remain. The protocol was not prospectively registered; searches were limited to two databases and English or Vietnamese reports; and one reviewer performed screening, extraction, and risk-of-bias assessment. Original database exports, complete bibliographic identities for excluded or unretrieved reports, and a component-by-component audit of all diagnostic implementations were not available for this revision. These documentation gaps limit reproducibility and leave search completeness and exact comparator harmonization open to verification. Exact sex-specific counts were reconstructed in some studies, paired covariance could not be modeled, and outcome-related evidence was sparse. In combination with five primary studies, extreme heterogeneity, and limited geographic coverage, these issues reduce confidence in the estimates and support hypothesis-generating rather than definitive conclusions.

## 5. Conclusions

This limited observational evidence base provides a structured, hypothesis-generating summary of sex-specific classification under IDF and NCEP-ATP III. The primary analysis showed no clear pooled difference in men and only a modest female excess under IDF that attenuated when revised ATP III was included. Five independent primary studies, extreme heterogeneity, and predominantly Asian study populations constrain robustness and generalizability. The estimates do not establish diagnostic equivalence, a sex-by-definition interaction, an epigenetic mechanism, prognostic superiority, or treatment benefit. Standardized reporting can coexist with population-appropriate thresholds, but their performance requires external validation. Explicit definitions, wider geographic representation, paired-data analyses, and prospective outcome comparisons are the main priorities arising from this evidence summary.

## Figures and Tables

**Figure 1:**
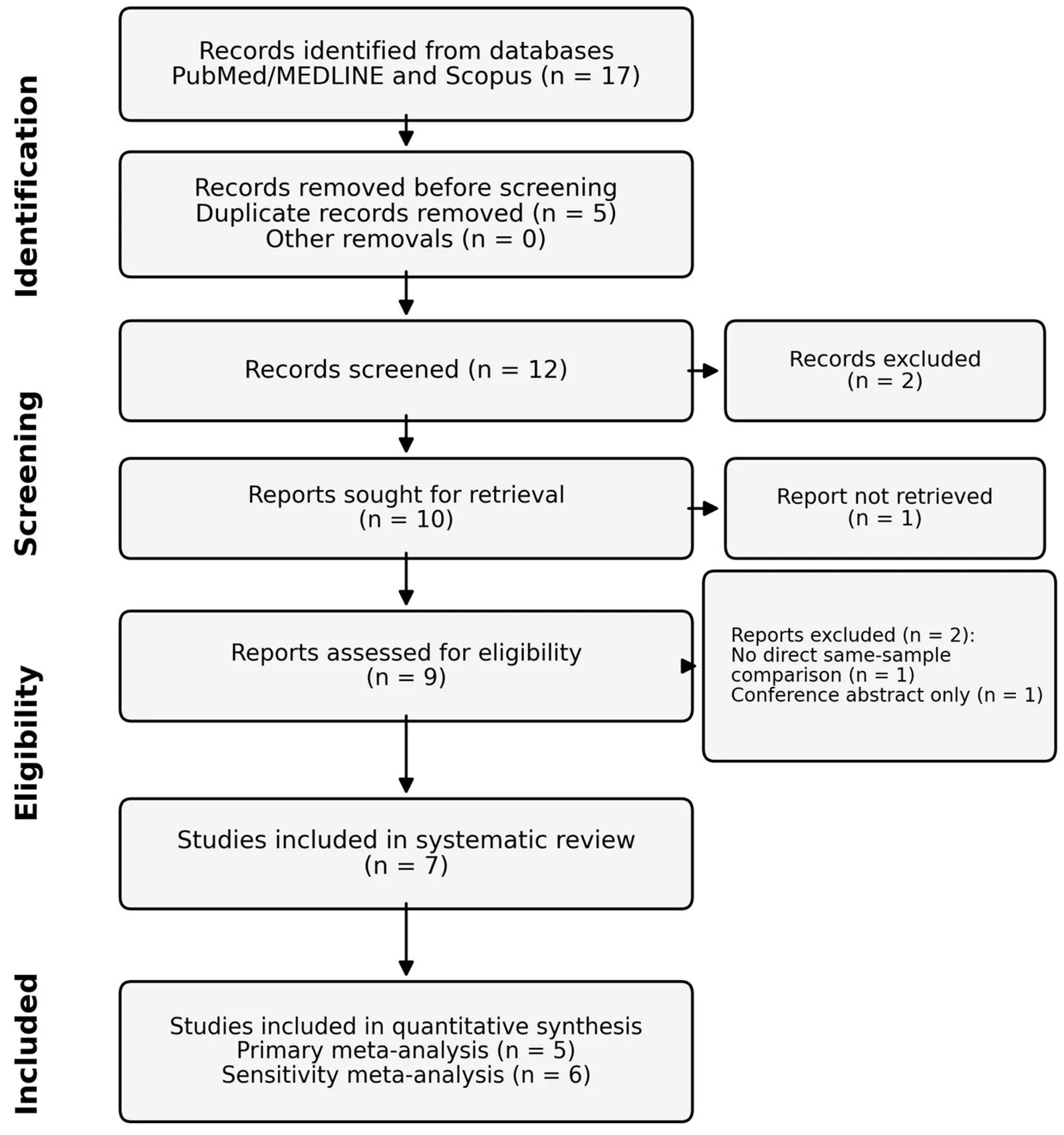
PRISMA 2020 flow diagram of study selection for searches reported through 30 August 2026. Counts are retained from the supplied screening summary. Five studies contributed to the strict primary meta-analysis; one additional study contributed to the ATP III-family sensitivity analysis. The one report not retrieved is separate from the two eligibility exclusions.

**Figure 2:**
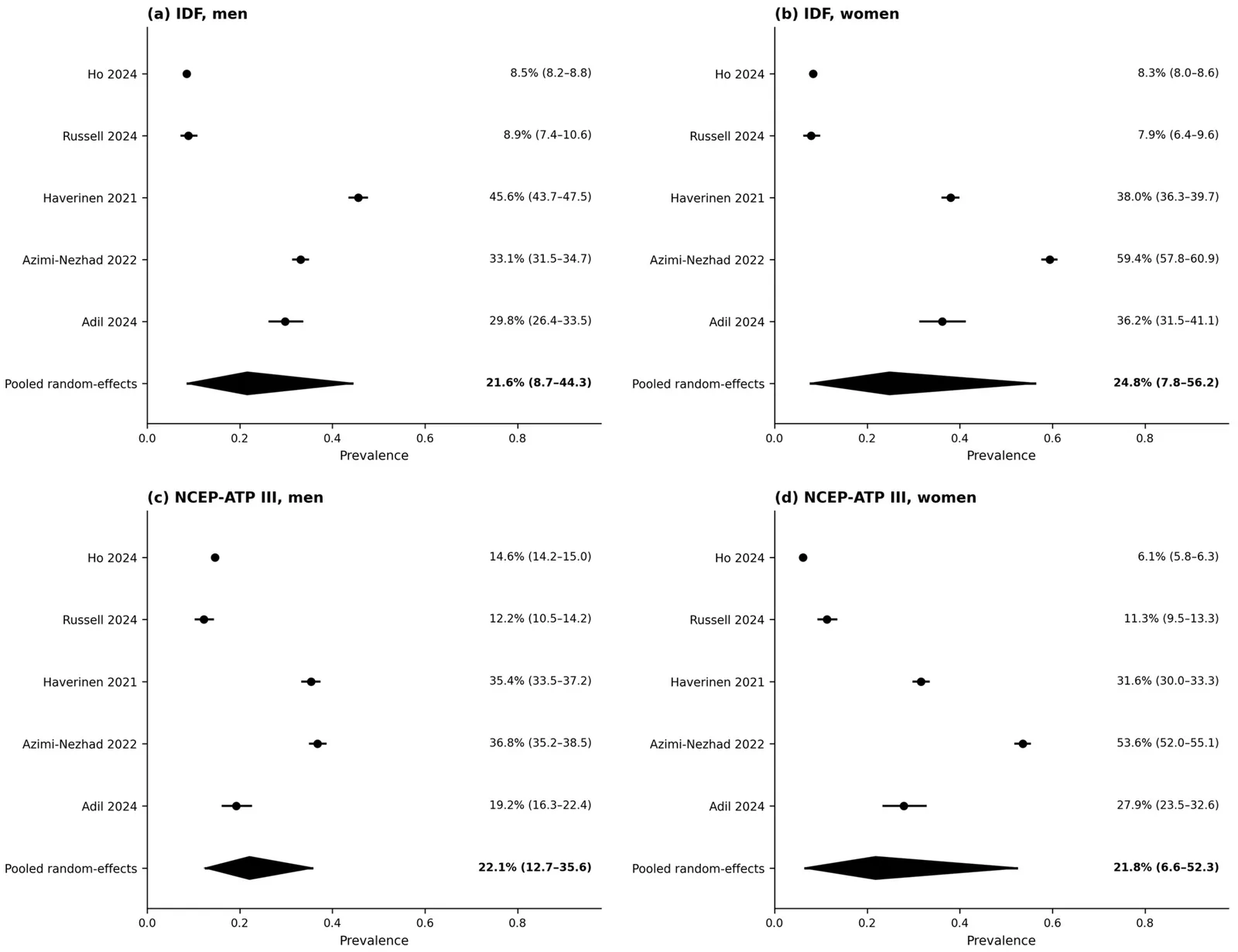
Sex-specific pooled prevalence of metabolic syndrome: (**a**) IDF in men; (**b**) IDF in women; (**c**) NCEP-ATP III in men; and (**d**) NCEP-ATP III in women. Diamonds denote pooled random-effects estimates. Studies shown from top to bottom: Ho et al., 2024 [10]; Russell et al., 2024 [15]; Haverinen et al., 2021 [13]; Azimi-Nezhad et al., 2022 [9]; and Adil et al., 2024 [14].

**Figure 3:**
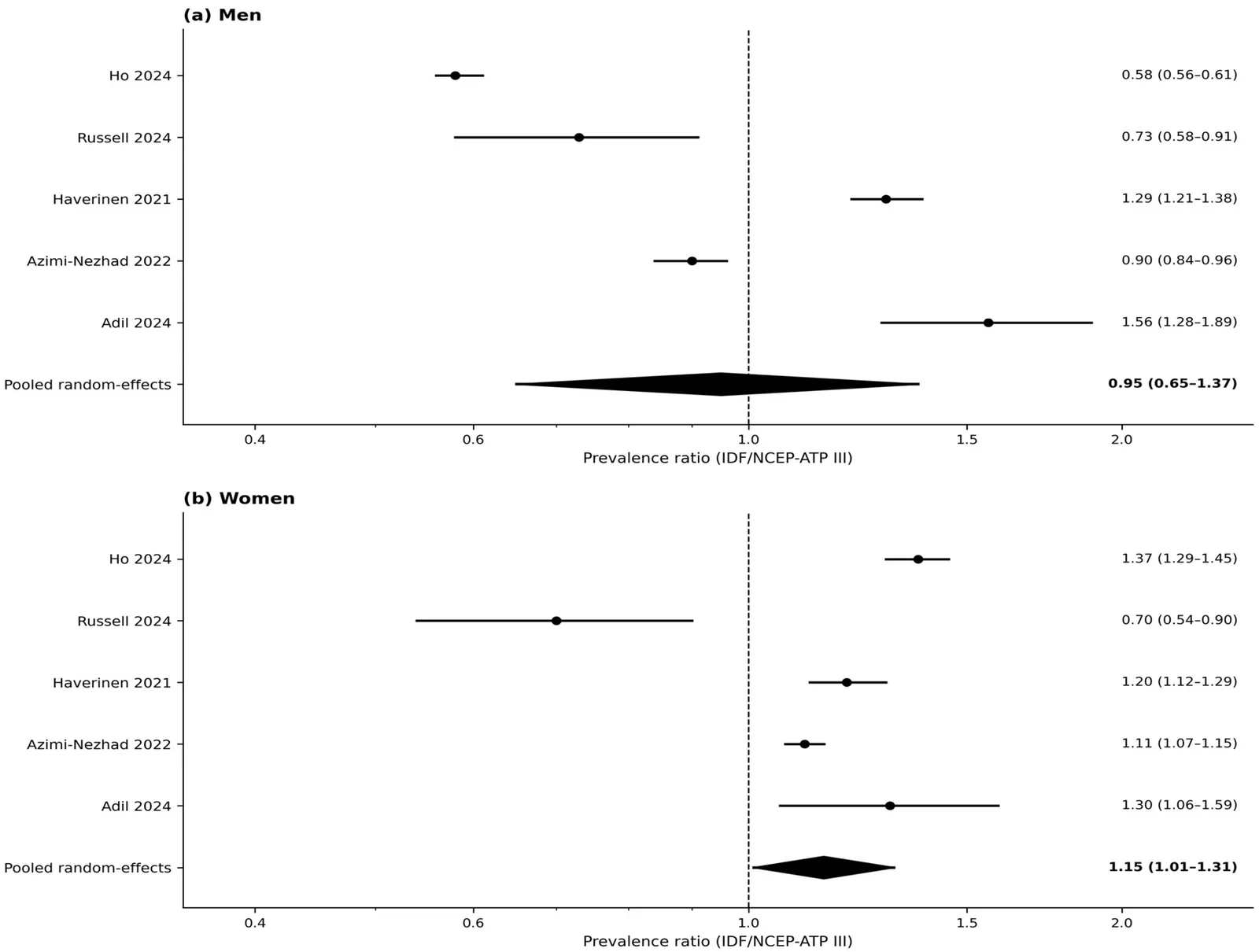
Head-to-head prevalence ratios (IDF/NCEP-ATP III) by sex. Ratios greater than 1 indicate higher classification under IDF; diamonds denote pooled random-effects estimates. Studies shown from top to bottom: Ho et al., 2024 [10]; Russell et al., 2024 [15]; Haverinen et al., 2021 [13]; Azimi-Nezhad et al., 2022 [9]; and Adil et al., 2024 [14].

**Table 1:** Characteristics of the included studies.

Study; Country	Design and Setting	Sample Size	Age Definition	Criteria Compared	Main Outcome Domain
Ho et al., 2024 [10]; Vietnam	Cross-sectional secondary analysis; occupational health checks	57,997	18–79 years	IDF; NCEP-ATP III; NCEP-ATP III-Asia	Overall and sex/age-specific prevalence; associated factors
Yousefzadeh et al., 2024 [11]; Iran	Analytical cross-sectional study; community-derived cohort sample	600	40–79 years	IDF; NCEP-ATP III	ASCVD risk discrimination; prevalence; agreement
Russell et al., 2024 [15]; Sierra Leone	Community-based cross-sectional urban survey	2394	≥20 years	NCEP-ATP III; IDF; harmonized ATP III	Prevalence; kappa agreement; correlates
Haverinen et al., 2021 [13]; Finland	Population-based cross-sectional national health examination survey	5687	≥25 years	WHO; NCEP-ATP III; IDF; JIS	Sex/age-specific prevalence; kappa agreement
Azimi-Nezhad et al., 2022 [9]; Iran	Population-based cross-sectional ageing-cohort baseline	7256	≥50 years	NCEP-ATP III; IDF	Sex-specific prevalence; correlates; kappa agreement
Huang et al., 2023 [12]; China	Prospective national cohort with follow-up	20,888	Adults ≥ 18 years; analytic cohort ≥35 years	Revised ATP III; IDF; JCDCG	Incident CVD/CHD/stroke; sex-specific discrimination
Adil et al., 2024 [14]; Pakistan	Community-based cross-sectional screening-camp survey	1065	25–80 years	NCEP-ATP III; IDF; modified NCEP-ATP III	Prevalence; associated factors; agreement

Abbreviations: ASCVD, atherosclerotic cardiovascular disease; CHD, coronary heart disease; CVD, cardiovascular disease; JCDCG, Joint Committee for Developing Chinese Guidelines; JIS, Joint Interim Statement; WHO, World Health Organization.

**Table 2:** Risk-of-bias assessment of the included studies.

Study	Sampling Frame	Recruitment	Definition Measurement	Outcome Ascertainment	Confounding Control	Statistical Analysis	Overall Risk
Ho et al., 2024 [10]	Moderate	Moderate	Low	Low	Moderate	Low	Moderate
Yousefzadeh et al., 2024 [11]	High	High	Low	Moderate	High	Moderate	High
Russell et al., 2024 [15]	Moderate	Low	Low	Low	Moderate	Low	Moderate
Haverinen et al., 2021 [13]	Low	Low	Moderate	Low	Low	Low	Low
Azimi-Nezhad et al., 2022 [9]	Low	Low	Low	Low	Moderate	Low	Low
Huang et al., 2023 [12]	Low	Low	Low	Low	Low	Low	Low
Adil et al., 2024 [14]	Moderate	Moderate	Low	Low	Moderate	Moderate	Moderate

**Table 3:** Primary and sensitivity quantitative syntheses.

Analysis	Studies	Participants	Pooled Estimate	95% CI	I^2^ (%)
IDF prevalence in men (primary)	5	36,033	21.6%	8.7–44.3	99.9
IDF prevalence in women (primary)	5	38,366	24.8%	7.8–56.2	99.9
NCEP-ATP III prevalence in men (primary)	5	36,033	22.1%	12.7–35.6	99.7
NCEP-ATP III prevalence in women (primary)	5	38,366	21.8%	6.6–52.3	99.9
IDF/NCEP-ATP III prevalence ratio in men (primary)	5	36,033	0.95	0.65–1.37	99.1
IDF/NCEP-ATP III prevalence ratio in women (primary)	5	38,366	1.15	1.01–1.31	92.3
IDF/ATP III-family prevalence ratio in men (sensitivity)	6	45,746	0.91	0.70–1.19	98.8
IDF/ATP III-family prevalence ratio in women (sensitivity)	6	49,541	1.08	0.92–1.27	97.5

CI, confidence interval; I^2^, percentage of total variability attributable to between-study heterogeneity. The ATP III-family sensitivity analysis added one study using revised ATP III.

## Data Availability

Study-level characteristics and numerical estimates are provided in Supplementary File S1. Supplementary File S2 provides the search syntax available for this revision and its documentation status (Table S6), eligibility criteria and available report-disposition information (Table S7), descriptive calculations of the geographic representation of the primary-analysis sample (Table S8), and selected historical milestones in the development of metabolic syndrome definitions (Table S9). Original database exports, a complete bibliographic screening log, and the original analysis environment were not supplied for independent verification during this revision. Requests concerning the underlying review records should be directed to the corresponding author.

## Supplementary Materials

The following supporting information can be downloaded at: https://www.mdpi.com/article/10.3390/metabo16100701/s1, Supplementary File S1 contains Tables S1–S5 (study characteristics and synthesis roles; risk-of-bias judgments; sex-specific prevalence estimates; prevalence ratios; and primary/sensitivity summaries). Supplementary File S2 contains Tables S6–S9 (available search syntax and documentation status; eligibility and report dispositions; geographic representation; and selected historical milestones in the development of MetS definitions).

## Abbreviations

The following abbreviations are used in this manuscript:

ASCVD	Atherosclerotic cardiovascular disease
CHD	Coronary heart disease
CI	Confidence interval
CVD	Cardiovascular disease
HDL-C	High-density lipoprotein cholesterol
IDF	International Diabetes Federation
JBI	Joanna Briggs Institute
JCDCG	Joint Committee for Developing Chinese Guidelines
JIS	Joint Interim Statement
MetS	Metabolic syndrome
NCEP-ATP III	National Cholesterol Education Program Adult Treatment Panel III
PRISMA	Preferred Reporting Items for Systematic Reviews and Meta-Analyses
ROC	Receiver operating characteristic
AHA	American Heart Association
NHLBI	National Heart, Lung, and Blood Institute
WHO	World Health Organization

## References

[1] Expert Panel on Detection, Evaluation, and Treatment of High Blood Cholesterol in Adults (2001). Executive summary of the third report of the National Cholesterol Education Program (NCEP) Expert Panel on Detection, Evaluation, and Treatment of High Blood Cholesterol in Adults (Adult Treatment Panel III). JAMA.

[2] Alberti K.G.M.M., Zimmet P., Shaw J. (2005). For the IDF Epidemiology Task Force Consensus Group. The metabolic syndrome—A new worldwide definition. Lancet.

[3] Reaven G.M. (1988). Banting lecture 1988. Role of insulin resistance in human disease. Diabetes.

[4] Alberti K.G.M.M., Zimmet P.Z. (1998). Definition, diagnosis and classification of diabetes mellitus and its complications. Part 1: Diagnosis and classification of diabetes mellitus. Provisional report of a WHO Consultation. Diabet. Med..

[5] World Health Organization (1999). Definition, Diagnosis and Classification of Diabetes Mellitus and Its Complications: Report of a WHO Consultation. Part 1: Diagnosis and Classification of Diabetes Mellitus.

[6] International Diabetes Federation (2006). The IDF Consensus Worldwide Definition of the Metabolic Syndrome.

[7] Grundy S.M., Cleeman J.I., Daniels S.R., Donato K.A., Eckel R.H., Franklin B.A., Gordon D.J., Krauss R.M., Savage P.J., Smith S.C. (2005). Diagnosis and management of the metabolic syndrome: An American Heart Association/National Heart, Lung, and Blood Institute Scientific Statement. Circulation.

[8] Alberti K.G.M.M., Eckel R.H., Grundy S.M., Zimmet P.Z., Cleeman J.I., Donato K.A., Fruchart J.-C., James W.P.T., Loria C.M., Smith S.C. (2009). Harmonizing the metabolic syndrome: A Joint Interim Statement of the International Diabetes Federation Task Force on Epidemiology and Prevention; National Heart, Lung, and Blood Institute; American Heart Association; World Heart Federation; International Atherosclerosis Society; and International Association for the Study of Obesity. Circulation.

[9] Azimi-Nezhad M., Aminisani N., Ghasemi A., Rezaei Farimani A., Khorashadizadeh F., Mirhafez S.R., Hyde M., Shamshirgaran S.M. (2022). Sex-specific prevalence of metabolic syndrome in older adults: Results from the Neyshabur Longitudinal Study on Ageing, Iran. J. Diabetes Metab. Disord..

[10] Ho N.T., Tran M.T., Tran C.T.D., Vanderbloemen L., Pham T.T., Hoang L.B., Nguyenj Q.V., Dornb J., Trevisanb M., Shuk X.-O. (2024). Prevalence of metabolic syndrome among Vietnamese adult employees. Nutr. Metab. Cardiovasc. Dis..

[11] Yousefzadeh G., Sayyadi A., Najafipour H., Sabaghnejad V., Pezeshki S. (2024). Comparing the association of two metabolic syndrome definitions, NCEP ATP III and IDF, with the risk of developing atherosclerotic cardiovascular disease: An analytical cross-sectional study. Endocrinol. Diabetes Metab..

[12] Huang Y., Chen Z., Wang X., Zheng C., Shao L., Tian Y., Cao X., Tian Y., Gao R., Zhang L. (2023). Comparison of the three most commonly used metabolic syndrome definitions in the Chinese population: A prospective study. Metabolites.

[13] Haverinen E., Paalanen L., Palmieri L., Padron-Monedero A., Noguer-Zambrano I., Sarmiento Suarez R., Tolonen H., Joint Action on Health Information (InfAct) (2021). Comparison of metabolic syndrome prevalence using four different definitions: A population-based study in Finland. Arch. Public Health.

[14] Adil S.O., Musa K.I., Uddin F., Khan A., Khan I., Shakeel A., Shafique K., Islam M.A. (2024). Prevalence of undiagnosed metabolic syndrome using three different definitions and identifying associated risk factors among apparently healthy adults in Karachi, Pakistan: A cross-sectional survey in the year 2022. Arch. Public Health.

[15] Russell J.B.W., Koroma T.R., Sesay S., Samura S.K., Lakoh S., Bockarie A., Abiri O.T., Conteh V., Conteh S., Smith M. (2024). Prevalence and correlates of metabolic syndrome among adults in Freetown, Sierra Leone: A comparative analysis of NCEP ATP III, IDF and harmonized ATP III criteria. Int. J. Cardiol. Cardiovasc. Risk Prev..

[16] Page M.J., McKenzie J.E., Bossuyt P.M., Boutron I., Hoffmann T.C., Mulrow C.D., Shamseer L., Tetzlaff J.M., Akl E.A., Brennan S.E. (2021). The PRISMA 2020 statement: An updated guideline for reporting systematic reviews. BMJ.

[17] Munn Z., Moola S., Lisy K., Riitano D., Tufanaru C. (2015). Methodological guidance for systematic reviews of observational epidemiological studies reporting prevalence and cumulative incidence data. Int. J. Evid. Based Healthc..

[18] Deeks J.J., Higgins J.P.T., Altman D.G., McKenzie J.E., Veroniki A.A., Higgins J.P.T., Thomas J., Chandler J., Cumpston M., Li T., Page M.J., Welch V.A. (2024). Chapter 10: Analysing data and undertaking meta-analyses. Cochrane Handbook for Systematic Reviews of Interventions, Version 6.5.

