# Supplementary material for: Sex-Specific Differences in Metabolic Syndrome Classification Using IDF Versus NCEP-ATP III Criteria: A Systematic Review and Head-to-Head Meta-Analysis
Source: Metabolites. 2026 Sep 22;16(10):701. doi: 10.3390/metabo16100701 (PMC13706850; doi:10.3390/metabo16100701)
Supplement: Supplementary file 1 [file metabolites-16-00701-s001.zip › metabolites-4563435-Supplementary File S1.pdf]

Supplementary File S1

Table S1. Characteristics and role of the included studies

Study; country	Design and setting	Sample size	Age definition	Criteria compared	Main outcome domain	Role in review
Ho et al., 2024; Vietnam	Cross-sectional secondary analysis; occupational health checks	57,997	18–79 years	IDF; NCEP-ATP III; NCEP-ATP III-Asia	Overall and sex/age-specific prevalence; associated factors	Primary meta-analysis
Yousefzadeh et al., 2024; Iran	Analytical cross-sectional study; community-derived cohort sample	600	40–79 years	IDF; NCEP-ATP III	ASCVD risk discrimination; prevalence; agreement	Narrative synthesis
Russell et al., 2024; Sierra Leone	Community-based cross-sectional urban survey	2,394	≥20 years	NCEP-ATP III; IDF; harmonized ATP III	Prevalence; kappa agreement; correlates	Primary meta-analysis
Haverinen et al., 2021; Finland	Population-based cross-sectional national health examination survey	5,687	≥25 years	WHO; NCEP-ATP III; IDF; JIS	Sex/age-specific prevalence; kappa agreement	Primary meta-analysis
Azimi-Nezhad et al., 2022; Iran	Population-based cross-sectional ageing-cohort baseline	7,256	≥50 years	NCEP-ATP III; IDF	Sex-specific prevalence; correlates; kappa agreement	Primary meta-analysis
Huang et al., 2023; China	Prospective national cohort with follow-up	20,888	Adults ≥18 years; analytic cohort ≥35 years	Revised ATP III; IDF; JCDCG	Incident CVD/CHD/stroke; sex-specific discrimination	Sensitivity and narrative synthesis
Adil et al., 2024; Pakistan	Community-based cross-sectional screening-camp survey	1,065	25–80 years	NCEP-ATP III; IDF; modified NCEP-ATP III	Prevalence; associated factors; agreement	Primary meta-analysis

Abbreviations: ASCVD, atherosclerotic cardiovascular disease; CHD, coronary heart disease; CVD, cardiovascular disease; IDF, International Diabetes Federation; JCDCG, Joint Committee for Developing Chinese Guidelines; JIS, Joint Interim Statement; NCEP-ATP III, National Cholesterol Education Program Adult Treatment Panel III; WHO, World Health Organization.

Table S2. Domain-level risk-of-bias assessment

Study	Sampling frame	Recruitment	Definition measurement	Outcome ascertainment	Confounding control	Statistical analysis	Overall risk
Ho et al., 2024	Moderate	Moderate	Low	Low	Moderate	Low	Moderate
Yousefzadeh et al., 2024	High	High	Low	Moderate	High	Moderate	High
Russell et al., 2024	Moderate	Low	Low	Low	Moderate	Low	Moderate
Haverinen et al., 2021	Low	Low	Moderate	Low	Low	Low	Low
Azimi-Nezhad et al., 2022	Low	Low	Low	Low	Moderate	Low	Low
Huang et al., 2023	Low	Low	Low	Low	Low	Low	Low
Adil et al., 2024	Moderate	Moderate	Low	Low	Moderate	Moderate	Moderate

The risk-of-bias assessments in this table are also presented in Table 2 of the main manuscript and are reproduced here for completeness of the supplementary materials.

Table S3. Study-level sex-specific prevalence estimates used in Figure 2

Study	Sex	Definition	Prevalence (%)	95% CI (%)
Ho et al., 2024	Men	IDF	8.5	8.2–8.8
Russell et al., 2024	Men	IDF	8.9	7.4–10.6
Haverinen et al., 2021	Men	IDF	45.6	43.7–47.5
Azimi-Nezhad et al., 2022	Men	IDF	33.1	31.5–34.7
Adil et al., 2024	Men	IDF	29.8	26.4–33.5
Ho et al., 2024	Women	IDF	8.3	8.0–8.6
Russell et al., 2024	Women	IDF	7.9	6.4–9.6
Haverinen et al., 2021	Women	IDF	38.0	36.3–39.7
Azimi-Nezhad et al., 2022	Women	IDF	59.4	57.8–60.9
Adil et al., 2024	Women	IDF	36.2	31.5–41.1
Ho et al., 2024	Men	NCEP-ATP III	14.6	14.2–15.0
Russell et al., 2024	Men	NCEP-ATP III	12.2	10.5–14.2
Haverinen et al., 2021	Men	NCEP-ATP III	35.4	33.5–37.2
Azimi-Nezhad et al., 2022	Men	NCEP-ATP III	36.8	35.2–38.5
Adil et al., 2024	Men	NCEP-ATP III	19.2	16.3–22.4
Ho et al., 2024	Women	NCEP-ATP III	6.1	5.8–6.3
Russell et al., 2024	Women	NCEP-ATP III	11.3	9.5–13.3
Haverinen et al., 2021	Women	NCEP-ATP III	31.6	30.0–33.3
Azimi-Nezhad et al., 2022	Women	NCEP-ATP III	53.6	52.0–55.1
Adil et al., 2024	Women	NCEP-ATP III	27.9	23.5–32.6

Table S4. Study-level head-to-head prevalence ratios used in Figure 3

Study	Sex	IDF/NCEP-ATP III prevalence ratio	95% CI
Ho et al., 2024	Men	0.58	0.56–0.61
Russell et al., 2024	Men	0.73	0.58–0.91
Haverinen et al., 2021	Men	1.29	1.21–1.38
Azimi-Nezhad et al., 2022	Men	0.90	0.84–0.96
Adil et al., 2024	Men	1.56	1.28–1.89
Pooled random-effects	Men	0.95	0.65–1.37
Ho et al., 2024	Women	1.37	1.29–1.45
Russell et al., 2024	Women	0.70	0.54–0.90
Haverinen et al., 2021	Women	1.20	1.12–1.29
Azimi-Nezhad et al., 2022	Women	1.11	1.07–1.15
Adil et al., 2024	Women	1.30	1.06–1.59
Pooled random-effects	Women	1.15	1.01–1.31

Table S5. Summary of the primary and sensitivity syntheses

Analysis	Studies	Participants	Pooled estimate	95% CI	I^2 (%)
IDF prevalence in men (primary)	5	36,033	21.6%	8.7–44.3	99.9
IDF prevalence in women (primary)	5	38,366	24.8%	7.8–56.2	99.9
NCEP-ATP III prevalence in men (primary)	5	36,033	22.1%	12.7–35.6	99.7
NCEP-ATP III prevalence in women (primary)	5	38,366	21.8%	6.6–52.3	99.9
IDF/NCEP-ATP III prevalence ratio in men (primary)	5	36,033	0.95	0.65–1.37	99.1
IDF/NCEP-ATP III prevalence ratio in women (primary)	5	38,366	1.15	1.01–1.31	92.3
IDF/ATP III-family prevalence ratio in men (sensitivity)	6	45,746	0.91	0.70–1.19	98.8
IDF/ATP III-family prevalence ratio in women (sensitivity)	6	49,541	1.08	0.92–1.27	97.5
