# Supplementary material for: Sex-Specific Differences in Metabolic Syndrome Classification Using IDF Versus NCEP-ATP III Criteria: A Systematic Review and Head-to-Head Meta-Analysis
Source: Metabolites. 2026 Sep 22;16(10):701. doi: 10.3390/metabo16100701 (PMC13706850; doi:10.3390/metabo16100701)
Supplement: Supplementary file 1 [file metabolites-16-00701-s001.zip › metabolites-4563435-Supplementary File S2.pdf]

Supplementary File S2

Table S6. Search syntax and documentation status

S6A. PubMed/MEDLINE

Field	Information
Platform	PubMed, U.S. National Library of Medicine
Reported final search date	30 August 2026
Intended coverage	Database inception through the reported final search date; no 2021 lower publication-year limit.
Fields searched	MeSH and Title/Abstract, as shown below.
Provenance	Reporting reconstruction supplied during preparation of the review materials; not independently verified against a time-stamped executed search history.
Filters and restrictions	The supplied Boolean string does not encode age, Humans, language, or date filters. Adult human populations and English/Vietnamese publication language are review eligibility criteria. Whether additional interface filters were applied historically is not documented.
Retrieval yield	Separate PubMed count unavailable. The manuscript reports a combined PubMed/Scopus total of 17 records before deduplication; this total has not been regenerated from original exports.

Available PubMed query

```
(("Metabolic Syndrome"[Mesh] OR
"metabolic syndrome"[Title/Abstract] OR MetS[Title/Abstract]) AND
("International Diabetes Federation"[Title/Abstract] OR IDF[Title/Abstract]) AND
("National Cholesterol Education Program"[Title/Abstract] OR
"NCEP ATP III"[Title/Abstract] OR "NCEP-ATP III"[Title/Abstract] OR
"Adult Treatment Panel III"[Title/Abstract] OR "ATP III"[Title/Abstract]) AND
(compar*[Title/Abstract] OR agreement[Title/Abstract] OR
concordance[Title/Abstract] OR prevalence[Title/Abstract] OR
sex[Title/Abstract] OR gender[Title/Abstract] OR women[Title/Abstract] OR
men[Title/Abstract] OR performance[Title/Abstract]))
```

The query above is not evidence that this exact string, with particular interface filters, returned 17 records on the reported date. The original database history and export are required to verify that linkage.

Table S6 (continued). Scopus and supplementary searching

S6B. Scopus

Field	Information
Platform	Scopus, Elsevier
Reported final search date	30 August 2026
Intended coverage	Database inception through the reported final search date.
Fields searched	TITLE-ABS-KEY (title, abstract, and keywords).
Filters explicitly encoded	English or Vietnamese publication language. No age, human-population, or publication-date restriction is encoded in the supplied string.
Provenance and yield	Reporting reconstruction available for this revision. Separate Scopus yield and original executed search history were not supplied. Do not assign the combined total of 17 independently to each database.

Available Scopus query

```
TITLE-ABS-KEY(("metabolic syndrome" OR MetS) AND
("International Diabetes Federation" OR IDF) AND
("NCEP ATP III" OR "NCEP-ATP III" OR "Adult Treatment Panel III" OR "ATP III") AND
(compar* OR agreement OR concordance OR prevalence OR "sex-specific" OR
sex OR gender OR women OR men OR performance)) AND
(LIMIT-TO(LANGUAGE, "English") OR LIMIT-TO(LANGUAGE, "Vietnamese"))
```

S6C. Reference-list screening and citation tracking

The manuscript reports reference-list screening and citation tracking as supplementary search methods. A source-specific record log, platform details, execution dates, and separate yields were not supplied. No additional record count, including a count of zero, is inferred for these methods.

Reconciliation of the reported counts

The retained screening summary reports 17 database records, five duplicates, 12 screened records, two title/abstract exclusions, 10 reports sought, one report not retrieved, nine reports assessed, two eligibility exclusions, and seven included studies. These counts are internally arithmetically consistent but are not independently validated search yields. A repeat search would need its actual execution date and a newly documented screening trail.

Table S7. Eligibility and report dispositions

S7A. Scope and exclusion rules

Criterion	Operational clarification
Population	Adults or older adults. Exclusively pediatric studies were outside scope. A pre-existing MetS diagnosis was not required.
Design and purpose	Original cross-sectional, cohort, or case-control observations comparing classifications or associations. Intervention effects alone were outside scope, even when participants had MetS.
Same-sample comparison	Both IDF and NCEP-ATP III had to be applied within the same underlying sample, or a direct comparison had to be reconstructable. A single-definition report without such a comparison was excluded.
Extractable information	At least one eligible prevalence, agreement, or downstream association outcome was required. An abstract-only report with insufficient information was excluded.
Publication language	English or Vietnamese. Other publication languages were outside the stated eligibility criteria.
Originality and duplication	Reviews, editorials, and other reports without original eligible observations were outside scope. Duplicate datasets without additional eligible information were not counted as independent evidence.
Primary versus broader review	Primary pooling additionally required the designated NCEP-ATP III comparator and extractable sex-specific prevalence. Revised ATP III and otherwise relevant reports lacking primary-pooling data could remain in sensitivity/narrative synthesis.
Retrieval failure	A report not obtained was recorded separately from an assessed report excluded on eligibility grounds.

These clarifications make the stated review scope explicit; they are not a claim that an additional round of record-level screening was conducted during this revision.

Study-level distinction

Five studies contributed to the primary synthesis: Ho, Russell, Haverinen, Azimi-Nezhad, and Adil. Huang contributed to the revised ATP III sensitivity and narrative analyses. Yousefzadeh contributed to narrative synthesis only. The latter two studies were not excluded from the systematic review (source: submitted Supplementary File S1, Table S1).

Table S7 (continued). Available record-disposition information

Presentation ID	Stage and count	Available reason	Bibliographic identity
NR-01	Report sought but not retrieved; n = 1	Full report could not be retrieved. Not counted among the two assessed-report eligibility exclusions.	Not available in the supplied materials.
FT-EX-01	Assessed report excluded; n = 1	No direct same-sample IDF versus NCEP-ATP III comparison.	Not available in the supplied materials.
FT-EX-02	Assessed report excluded; n = 1	Conference abstract only; insufficient information for eligibility verification and extraction.	Not available in the supplied materials.

The IDs above are presentation labels assigned for this reporting table, not recovered identifiers from an original screening database. Authors, titles, publication years, and DOI/PMID information cannot be inferred from exclusion reasons. PRISMA item 16b remains incompletely verifiable until the two excluded reports are identified from the original screening records.

Source: submitted manuscript, Section 3.1 and Figure 1. The available record describes nine assessed reports, one of which was an abstract-only report; accordingly, the revision uses "reports assessed for eligibility" rather than implying that nine complete full-text articles were available.

Table S8. Geographic composition of the primary synthesis

Study	Country	Participants	Share of primary sample
Ho et al., 2024	Vietnam	57,997	78.0%
Azimi-Nezhad et al., 2022	Iran	7,256	9.8%
Adil et al., 2024	Pakistan	1,065	1.4%
Haverinen et al., 2021	Finland	5,687	7.6%
Russell et al., 2024	Sierra Leone	2,394	3.2%
Primary-analysis total	Five studies	74,399	100.0%
Studies in Asian countries	Vietnam + Iran + Pakistan	66,318	89.1%

Calculation: (57,997 + 7,256 + 1,065) / 74,399 × 100 = 89.1%; 57,997 / 74,399 × 100 = 78.0%. Percentages are rounded to one decimal. Source: submitted Supplementary File S1, Table S1. These are descriptive sample-composition calculations, not new pooled effect estimates.

Country of recruitment is not individual ethnicity. Participant shares are not inverse-variance random-effects weights. China is represented in the sensitivity analysis, not among the five primary studies; the second Iranian study is narrative only.

Table S9. Selected historical milestones in the development of metabolic syndrome definitions

Contextual narrative overview for Reviewer 2; not additional eligible studies or a new systematic search. The dates below identify the cited historical publications, not the final date of the review search (30 August 2026).

Year	Milestone and sources	Main conceptual or operational feature	Relevance to interpreting this review
1988	Reaven: insulin-resistance concept [3]	Linked insulin resistance to clustered metabolic and cardiovascular abnormalities. This was a conceptual account of pathophysiology, not the later five-component threshold algorithm.	Distinguishes a proposed biological framework from an operational classification rule. Classification disagreement in this review does not test or validate the underlying mechanism.
1998–1999	WHO provisional report and consultation report [4,5]	Formalized MetS using impaired glucose regulation, diabetes and/or insulin resistance together with additional abnormalities. The 1999 report included raised blood pressure, dyslipidemia, obesity and microalbuminuria among those features.	Illustrates the earlier glucose/insulin-resistance-centered approach and its broader component set. These historical WHO definitions were not pooled as additional comparators in the present review.
2001	NCEP-ATP III [1]	Used any three of five routinely assessed components: waist circumference, triglycerides, HDL-C, blood pressure and fasting glucose. Original fasting glucose threshold: ≥110 mg/dL; central obesity was not mandatory.	Establishes the original reference definition. An ATP III label alone is insufficient to verify the glucose, waist and medication rules actually used in a later publication.
2005	IDF consensus; detailed consensus document published in 2006 [2,6]	Required central obesity using ethnic-group-specific waist thresholds, plus any two of four additional components. The glucose criterion was ≥100 mg/dL or previously diagnosed type 2 diabetes.	Both the mandatory waist component and its threshold can alter case classification. IDF was an alternative framework, not merely a renamed or uniformly superior ATP III algorithm.
2005	AHA/NHLBI scientific statement [7]	Retained any three of five components; used fasting glucose ≥100 mg/dL and recognized drug treatment for specified lipid, blood-pressure and glucose abnormalities.	Explains why revised ATP III-family implementations must be distinguished from the original 2001 definition. The present review retains its primary-versus-sensitivity distinction.

Year	Milestone and sources	Main conceptual or operational feature	Relevance to interpreting this review
2009	Joint interim statement / harmonized definition [8]	Agreed on any three of five abnormalities without a compulsory component. A common set of component cut points was proposed except for waist circumference, for which national or regional values could be used pending further work.	Shows that a shared diagnostic structure can coexist with population-adapted waist thresholds. Harmonization is not itself proof of equal prediction or treatment benefit across populations.

References [1-8] correspond to the numbered bibliography in the revised main manuscript. The table is selective rather than an exhaustive history.
